# Supplementary material for: Distribution of Embryonic Stem Cell-Derived Mesenchymal Stem Cells after Intravenous Infusion in Hypoxic–Ischemic Encephalopathy
Source: Life (Basel). 2023 Jan 13;13(1):227. doi: 10.3390/life13010227 (PMC9861288; doi:10.3390/life13010227)

Supine position image - Normal

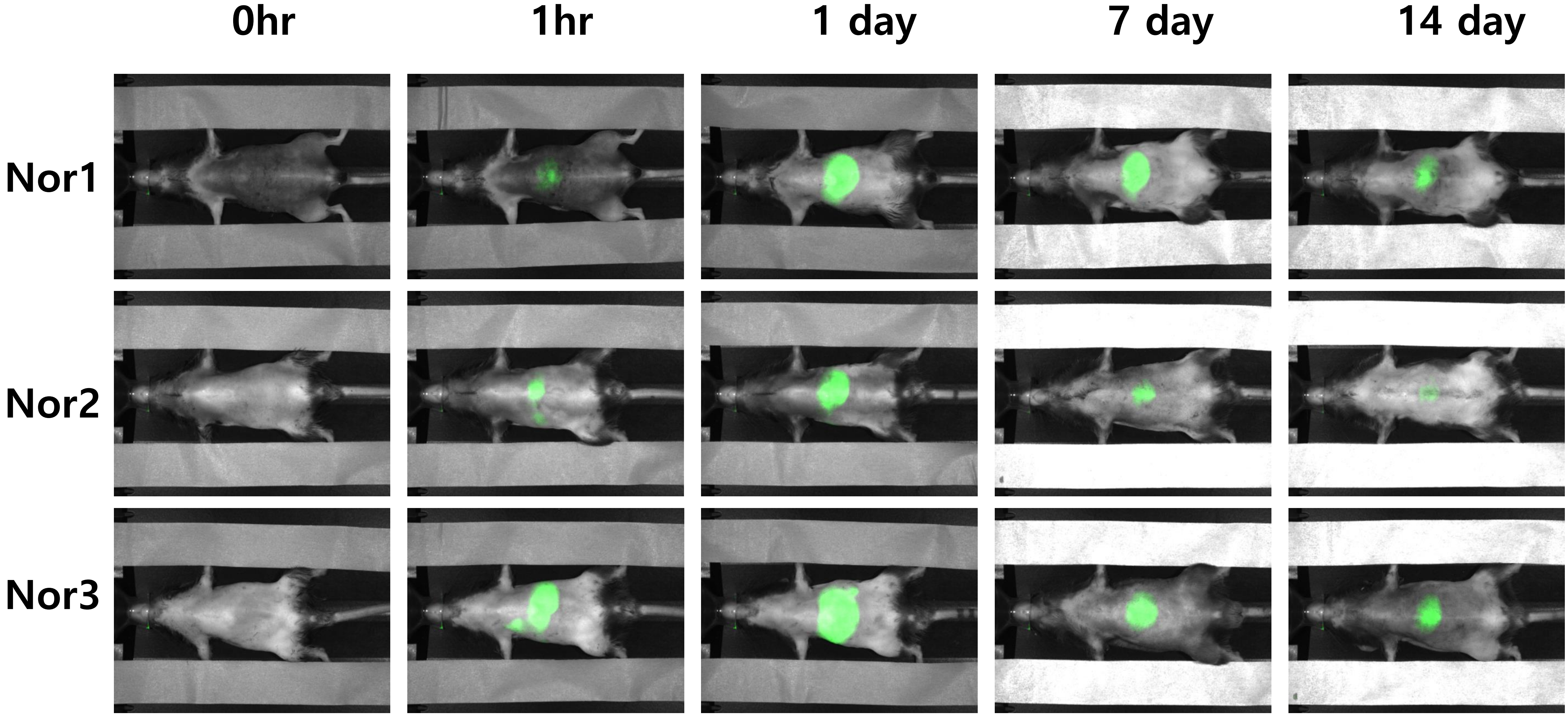

Prone position image - Normal

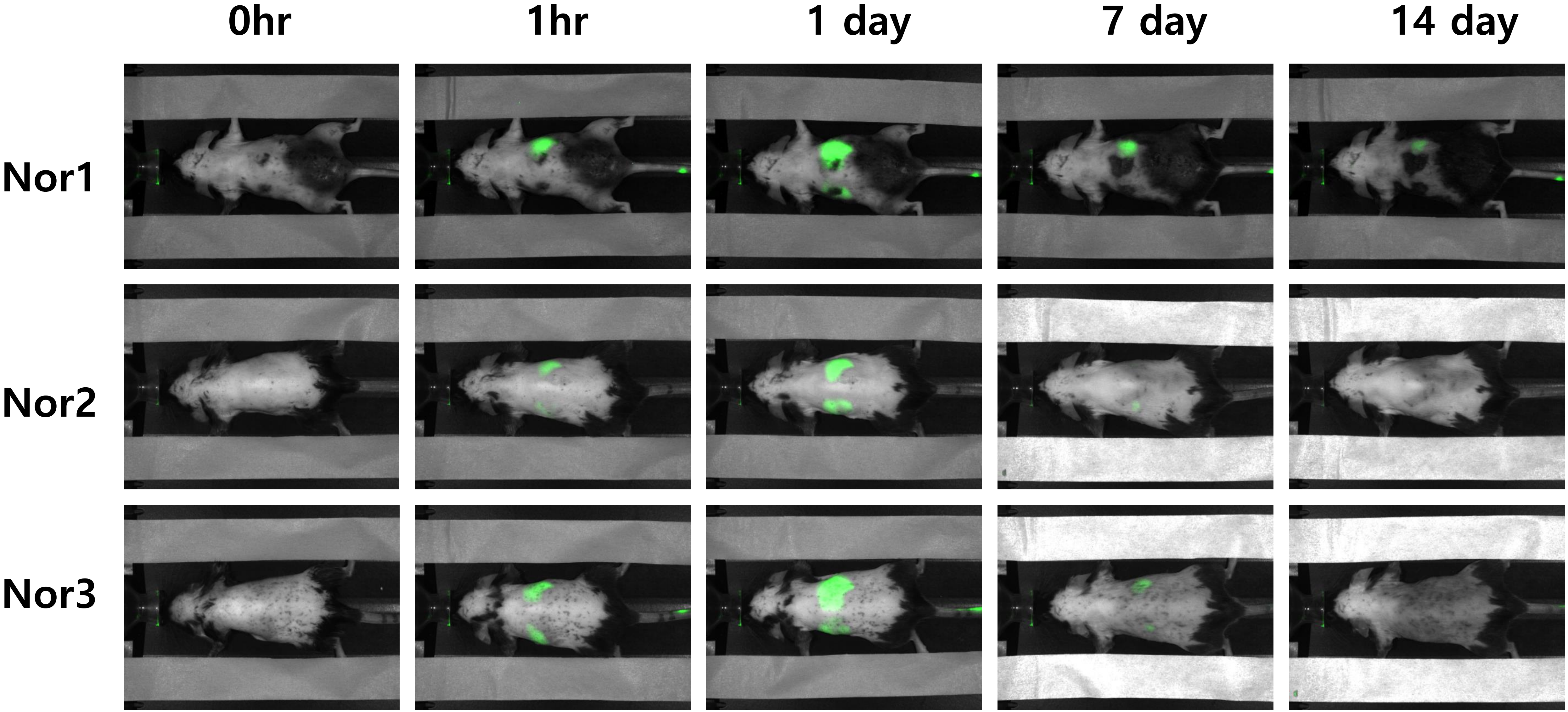

Prone position image (Brain) - Normal

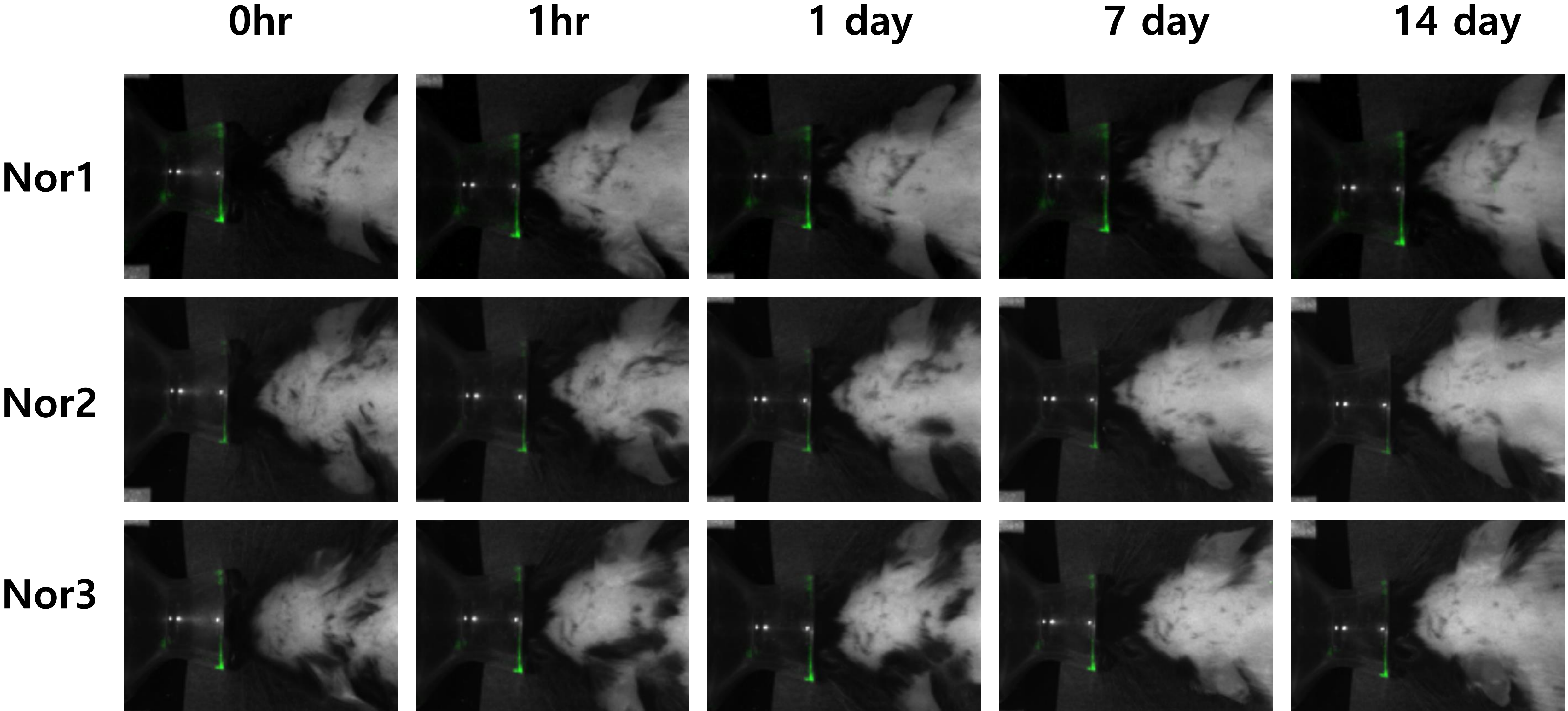

Supine position image - HIE

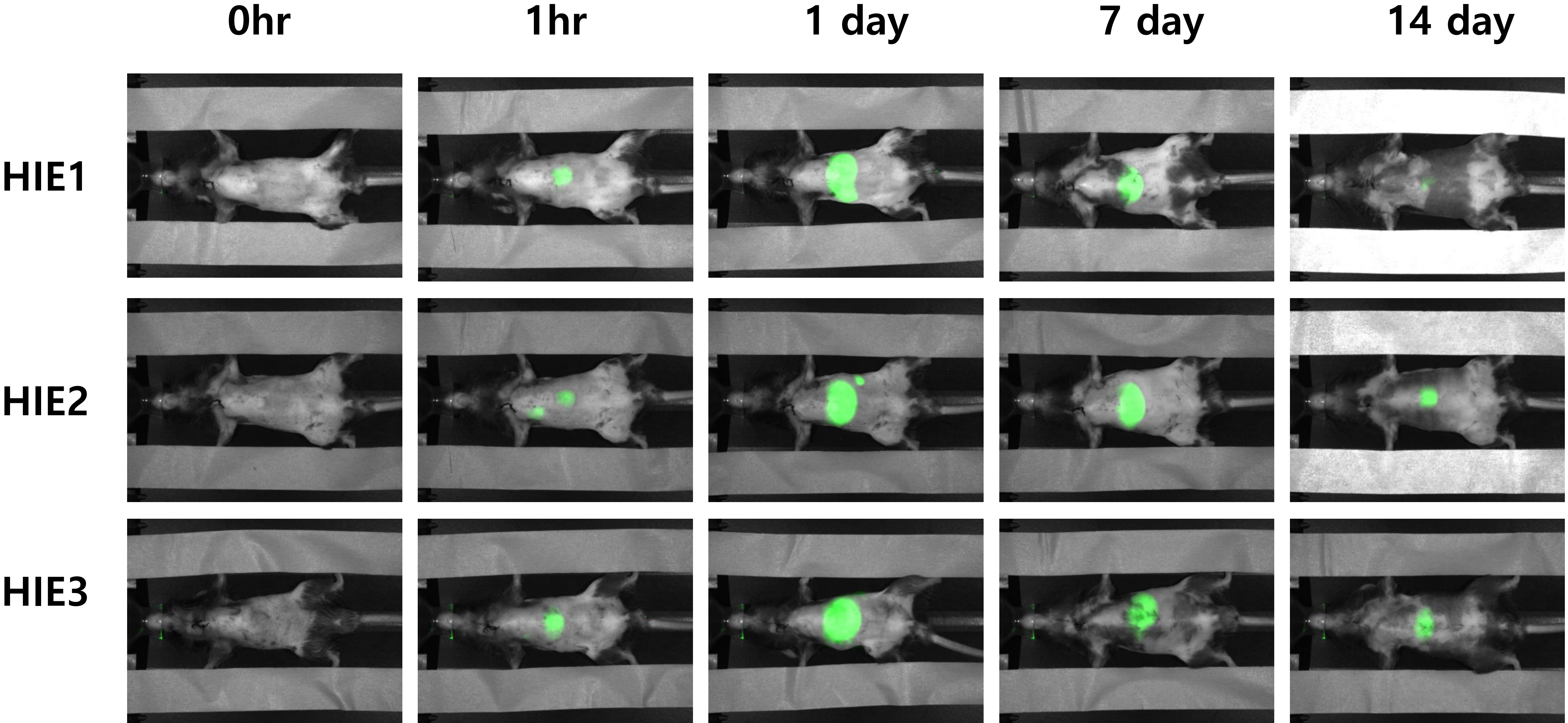

Prone position image - HIE

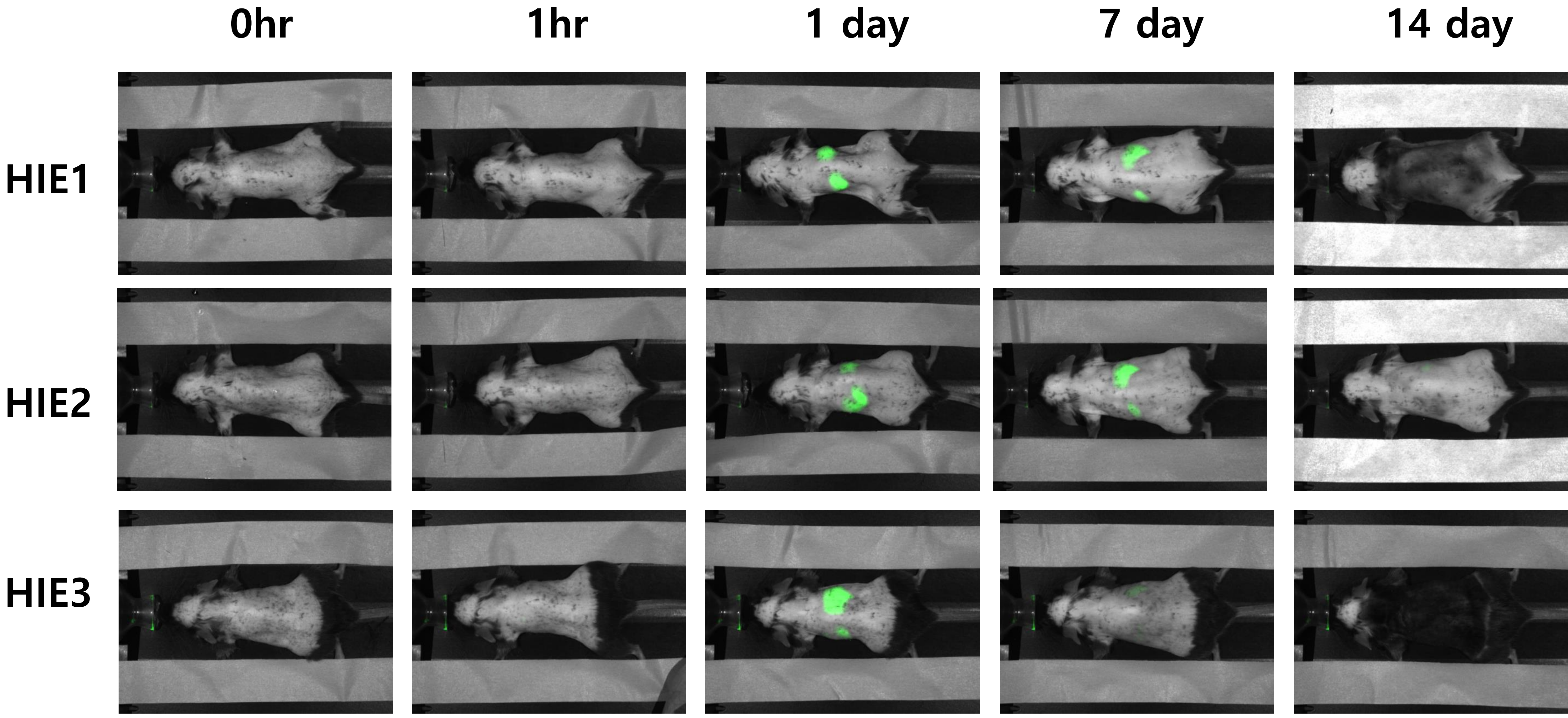

Prone position image (Brain) - HIE

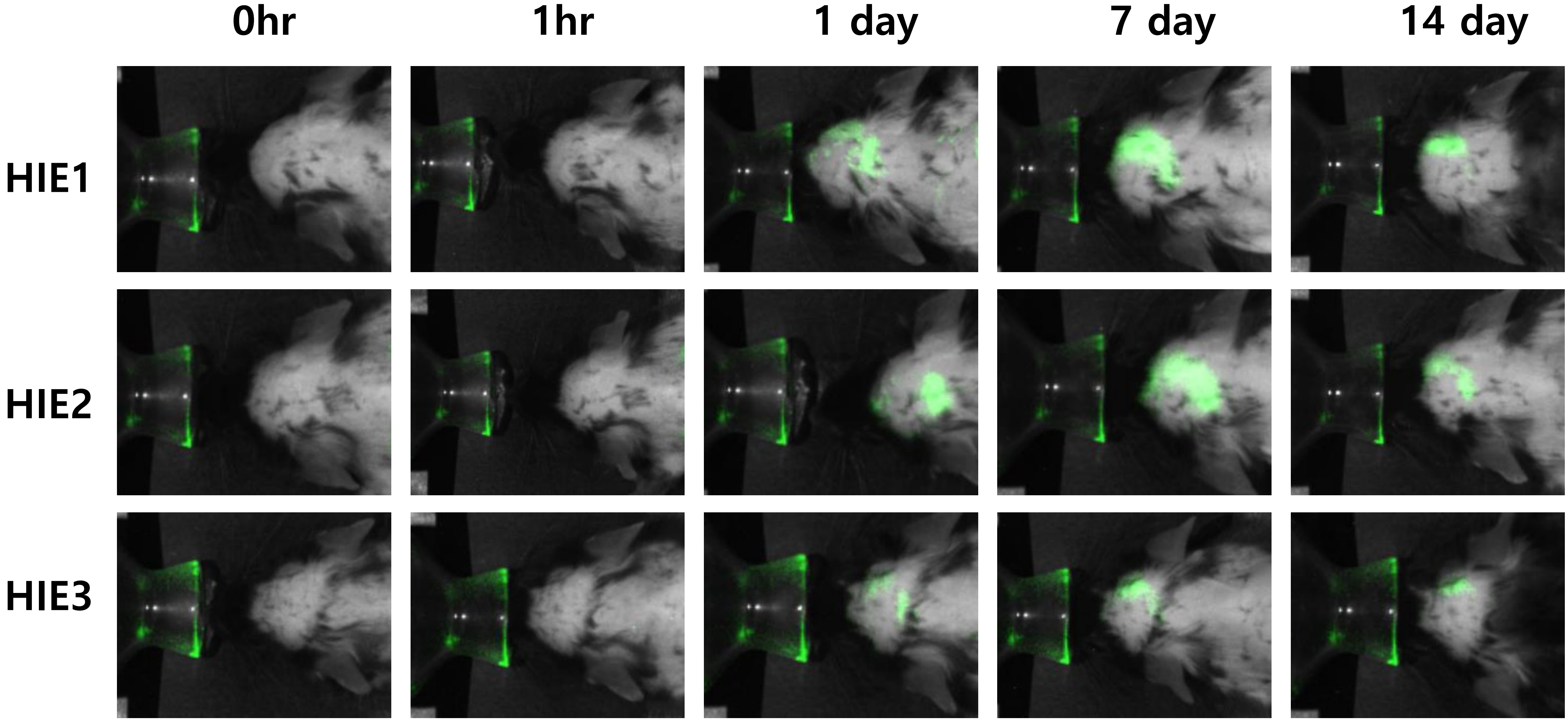

Supplement: Supplementary file 1 [file life-13-00227-s001.zip › life-2162137-supplementary.pdf]
